# Willingness to Communicate, L2 Self-Confidence, and Academic Self-Concept: A Mixed-Methods Study of Vietnamese University Students in the UK

**DOI:** 10.3390/bs15091176

**Published:** 2025-08-29

**Authors:** Ngo Nhat Thanh Tra, Weifeng Han, Shane Pill

**Affiliations:** 1 Faculty of Health, Education and Society, University of Northampton, Northampton NN1 5PH, UK; 2 College of Education, Psychology and Social Work, Flinders University, Adelaide 5042, Australia

**Keywords:** willingness to communicate (WTC), L2 self-confidence, academic self-concept, Vietnamese learners, mixed methods, second language acquisition, higher education, ESL context

## Abstract

The study investigates the interplay among second language (L2) learners’ willingness to communicate (WTC), L2 self-confidence (L2SC), and academic self-concept (ASC) within a cohort of Vietnamese university students studying in the United Kingdom. Employing an explanatory sequential mixed-methods design, self-reported quantitative data were collected via validated survey instruments (*n* = 32 students), followed by semi-structured interviews with a purposive subsample (*n* = 5 students) to contextualise the findings. Results revealed that participants exhibited moderate levels of WTC and L2 self-confidence, alongside emerging academic self-concept. Significant positive correlations were observed between WTC and L2 self-confidence and between L2 self-confidence and academic self-concept; a weak, non-significant association was found between WTC and academic self-concept. Qualitative data corroborated these patterns, highlighting how learners’ communicative confidence was shaped by supportive environments and evolving self-perceptions. While self-comparisons and cultural expectations occasionally influenced students’ academic self-concept, most participants reported resilience and a commitment to communication development. The study contributes to the literature by integrating psychological and contextual variables influencing WTC, offering pedagogical implications for enhancing communicative competence among international English as a second language learners.

## 1. Introduction

Despite the growing prominence of English in higher education across Asia, many Vietnamese learners continue to exhibit limited oral engagement, especially in cross-cultural contexts. While receptive proficiency remains high, a significant proportion (approximately 67%) report difficulties initiating or sustaining spoken interaction (Dinh & Tran, 2020). This behavioural gap underscores the need to examine psychological factors that mediate learners’ willingness to communicate, particularly among students studying abroad.

Reports within UK universities suggest that Vietnamese students, despite prior English exposure, often demonstrate hesitancy in engaging with international peers. This reluctance may stem less from linguistic deficits than from complex psychosocial factors, such as communicative anxiety (CA), self-perceived efficacy, and social identity concern, that shape interactional behaviours in multilingual settings. Despite the common belief that students studying abroad are proficient in English (Hessel, 2016; Hessel & Vanderplank, 2018; Li, 2014; Moore et al., 2021), many Vietnamese learners experience difficulties with verbal communication. Some learners, despite their intentions to broaden their social circles and engage with diverse groups, tend to gravitate towards familiar social settings where they can use their mother tongue, thus minimising English use and interaction.

**Figure A1:**
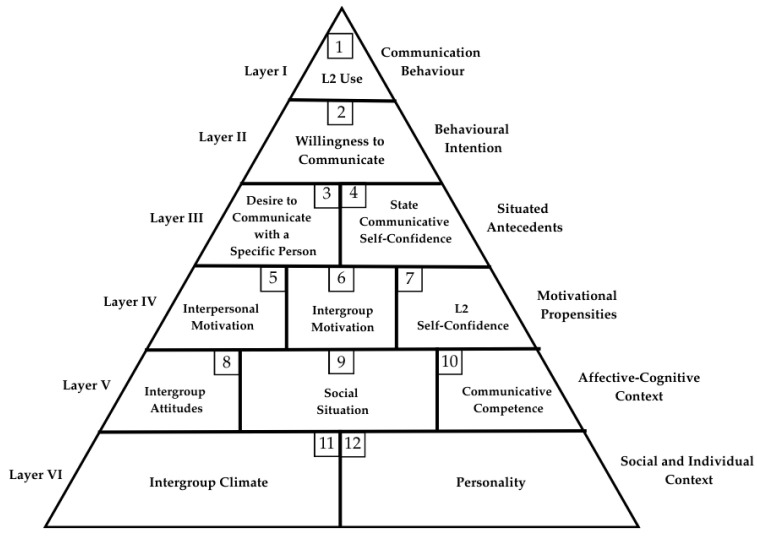
Heuristics Model of Variables Influencing L2 Willingness to Communicate (D. MacIntyre et al., 1998).

Under the Heuristics Model of variables influencing L2 WTC (Figure A1, D. MacIntyre et al., 1998), while L2 self-confidence was nested under the Motivational Propensities layer, academic self-concept was related to L2 self-confidence (Mercer, 2011). Prior studies have identified a constellation of variables, such as L2 self-confidence, academic self-concept, and communicative motivation, that influence WTC (D. MacIntyre et al., 1998; Amiryousefi & Mirkhani, 2019). However, empirical research has disproportionately focused on learners in Western or West Asian contexts—e.g., Iran and Turkey (Bektaş-Çetinkaya, 2005; Saka & Merç, 2021)—with relatively little attention to Southeast Asian students, particularly those studying abroad (Loan, 2019; Bui et al., 2022). This underrepresentation limits our understanding of how cultural and academic self-perceptions shape second language communication behaviour in English as a second language (ESL) Vietnamese students.

Because there is relatively little attention given to L2 WTC with Southeast Asian students studying abroad, this study aims to examine the interrelationships among Vietnamese international students’ willingness to communicate, L2 self-confidence, and academic self-concept in a United Kingdom (UK) university setting. By integrating behavioural and psychological variables within a mixed-methods framework, the study seeks to identify patterns that may inform targeted pedagogical and support strategies for international L2 learners.

This study addresses two research questions:

1. What are Vietnamese learners’ perceptions of WTC, academic self-concept, and L2 self-confidence?
2. What are the relationships among these three constructs?

## 2. Theoretical and Empirical Background

### 2.1. Overview of Key Constructs

#### 2.1.1. Academic Self-Concept

Academic self-concept refers to learners’ self-perceptions of competence and worth within academic contexts. It encompasses individuals’ beliefs about their abilities in specific domains and their evaluations of academic success (Bong & Skaalvik, 2003; Pajares & Schunk, 2005). Unlike self-esteem or general self-efficacy, academic self-concept is domain-specific and both cognitively and affectively oriented. In language learning, it influences how learners view themselves as communicators, particularly in high-stakes or culturally unfamiliar environments (Bong & Skaalvik, 2003; Byrne, 1984; Marsh & Craven, 2006; Wigfield & Karpathian, 1991). For international students studying in Western English first-language countries, academic self-concept plays a key behavioural role in shaping classroom participation, goal-setting, and persistence (Basileo et al., 2024; Yang et al., 2023).

#### 2.1.2. L2 Self-Confidence

L2 self-confidence comprises self-perceived communicative competence (SPCC) and language anxiety (Clément, 1980; DeVito, 1986; Fallah, 2014). L2 self-confidence refers to learners’ belief in their ability to communicate effectively in a second language (D. MacIntyre et al., 1998). Particularly, learners with high L2 self-confidence are expected to show strong belief in their L2 abilities and low levels of communication apprehension (Pattapong, 2015). For international students studying in Western English first-language countries, L2 self-confidence is also essential for maintaining meaningful conversation in their target language by using their L2 communication skills effectively and adaptively (Fatima et al., 2020; D. MacIntyre et al., 1998). Within ESL, more recent studies also claimed that a higher level of self-confidence would raise the probability of students being successful in English language studies (Yousefabadi & Ghafournia, 2023).

#### 2.1.3. Willingness to Communicate (WTC)

Willingness to communicate refers to a learner’s readiness to initiate communication in a second language, shaped by both enduring personality traits and dynamic situational factors (D. MacIntyre et al., 1998; McCroskey & Richmond, 1990). Trait WTC tends to be stable over time, whereas state WTC can shift depending on context (Peng, 2016). Understanding WTC offers insight into why some individuals actively engage in L2 interactions while others avoid them (McCroskey & Baer, 1985; McCroskey & Richmond, 1990, as cited in Kanat-Mutluoğlu, 2016). Two concepts are associated with WTC: communication apprehension, the anxiety associated with initiating conversation, and perceived communicative competence, or how capable learners feel in using the language (McCroskey, 1977, 1982; D. MacIntyre, 1994). Building on these two concepts, D. MacIntyre et al. (1998) proposed a heuristic model identifying multiple layers of influence on WTC, ranging from social and individual contexts to communicative confidence. This model has informed a range of subsequent studies (e.g., Katiandagho & Sengkey, 2022; Nadeem et al., 2023). In the context of international students studying in Western English-speaking countries, WTC is operationally defined in this study as the learner’s readiness to engage in spoken interaction. It is a central construct in examining their communicative behaviour in academic and social environments.

### 2.2. Theoretical Framework

The conceptual framework for this study draws primarily on D. MacIntyre et al.’s (1998) Heuristic Model of variables influencing L2 WTC, as well as extensions from the Theory of Planned Behaviour (Ajzen, 1988) and Mercer’s (2011) components of academic self-concept. Together, these frameworks clarify the pathways by which individual psychological factors and contextual influences interact to shape communication behaviour in L2 contexts.

D. MacIntyre et al.’s (1998) Heuristic Model positions WTC as the culmination of a dynamic, multilayered process involving both enduring personal dispositions and immediate situational factors. Central to this model is the notion that L2 self-confidence—comprising self-perceived communicative competence and communication anxiety—acts as a proximal antecedent to WTC. Self-confidence is, in turn, shaped by broader motivational propensities, including attitudes, social support, and contextual variables.

The Theory of Planned Behaviour (Ajzen, 1988) further illuminates the mechanisms underlying behavioural intention to communicate. According to this theory, a learner’s intention to engage in L2 communication is influenced by their attitudes towards the behaviour, perceived subjective norms, and perceived behavioural control. These components closely align with elements within D. MacIntyre et al.’s (1998) model, particularly in explaining how psychological readiness is translated into actual communicative action. While positive attitudes and supportive social norms foster intention, perceived control—related to self-confidence—determines whether that intention is realised in observable behaviour.

Mercer’s (2011) components of academic self-concept offer a complementary perspective, suggesting that academic self-concept is a higher-order construct encompassing self-confidence, self-efficacy, and self-related person knowledge. Within this definition, academic self-concept shapes learners’ beliefs about their capacity for educational and communicative success, thereby influencing both self-confidence and the propensity to engage in L2 communication. Mercer (2011) highlights that these self-perceptions are dynamic, context-sensitive, and co-constructed through social interaction—characteristics that are especially salient for international students navigating new cultural and linguistic environments.

Integrating these frameworks, the present study hypothesises a pathway in which academic self-concept influences L2 self-confidence, which in turn mediates willingness to communicate. This approach reflects the view that learners’ readiness to engage in second language interaction is a product of interrelated psychological, behavioural, and contextual factors, rather than a fixed trait or simple outcome of language proficiency.

By foregrounding these theoretical models, this study situates the investigation of Vietnamese students’ L2 communication behaviour within a robust and contemporary conceptual framework. This integration not only clarifies the anticipated relationships among WTC, L2 self-confidence, and academic self-concept but also establishes a foundation for examining how these constructs manifest in multicultural, English-medium university contexts.

### 2.3. Empirical Evidence Supporting Construct Relationships

#### 2.3.1. L2 Self-Confidence and Willingness to Communicate

Within D. MacIntyre et al.’s (1998) Heuristic Model of variables influencing L2 WTC, L2 self-confidence, comprised of self-perceived communicative competence and communication anxiety, is identified as a direct and pivotal antecedent to WTC. Learners who perceive themselves as competent and experience lower levels of communication anxiety are significantly more likely to initiate and sustain interaction in the target language (D. MacIntyre et al., 1998; Peng & Woodrow, 2010).

A substantial body of empirical research has established a robust positive association between L2 self-confidence and WTC across diverse ESL contexts (Bektaş-Çetinkaya, 2005; Ghonsooly et al., 2012; Hashimoto, 2002; Yashima, 2002). These findings support the notion that communication reluctance often stems from a lack of confidence (Alenezi, 2020; Fernando, 2022; Katiandagho & Sengkey, 2022; Peng & Woodrow, 2010). Studies also reveal that the underlying factors contributing to diminished self-confidence, including fear of negative evaluation, apprehension about making mistakes, and concerns about being ridiculed, can significantly impede learners’ willingness to communicate (Fernando, 2022).

#### 2.3.2. Academic Self-Concept and Willingness to Communicate

Empirical research increasingly suggests that academic self-concept (ASC), i.e., learners’ beliefs about their academic ability and self-worth, may play a nuanced role in shaping WTC in a second language (Pavlenko & Lantolf, 2000). Several studies have demonstrated that students with a positive ASC are generally more motivated to participate in classroom interactions and to take communicative risks, and view themselves as competent contributors (Lyons et al., 2014; Yue, 2016). For example, Yue (2016) found that students with higher ASC were more likely to see themselves as leaders or initiators in language learning, which in turn elevated their WTC, while those with less positive ASC reported greater hesitation and self-limiting behaviour.

However, the relationship is neither direct nor uniform. Research indicates that ASC can be destabilized by social comparison, cultural expectations, and prior academic experiences (Pattapong, 2015; Peng, 2016). For instance, in highly collectivist or “face-conscious” cultures, students may experience increased self-scrutiny and anxiety about peer evaluation, therefore, leading to fluctuations in their WTC even when their language proficiency is high (Leung, 1998; Pavlenko & Lantolf, 2000). Lyons et al. (2014) also highlight that a low or uncertain ASC, stemming from repeated negative feedback or perceived lack of ability, can dampen students’ willingness to communicate, sometimes even in otherwise supportive environments.

Taken together, these findings underscore that while a positive academic self-concept may support WTC by fostering self-assurance and engagement, its influence is context-dependent and can be moderated by social, cultural, and situational factors. Noticeably, there remains a need for further research in multilingual settings, especially among international students in English-speaking environments, to clarify how ASC operates alongside other psychological and behavioural constructs.

#### 2.3.3. Academic Self-Concept and L2 Self-Confidence

Although the direct relationship between ASC and L2 self-confidence has been less frequently examined in empirical research, theoretical models and a growing number of studies point to a meaningful association between the two. ASC provides a broad foundation of self-beliefs regarding one’s capabilities and adaptability within academic settings (Mercer, 2011; Bong & Skaalvik, 2003). L2 self-confidence, in contrast, is a more specific construct focused on perceived communicative competence and language-related anxiety (Clément, 1980; D. MacIntyre et al., 1998).

Theoretically, Mercer (2011) and Clément (1980) propose that ASC underpins the development of L2 self-confidence by shaping how learners interpret successes, setbacks, and feedback in the language-learning process. Learners who view themselves as capable and resourceful academically are likely to be more resilient in the face of communicative challenges, which demonstrates greater confidence in their L2 abilities. Empirical work by Lubis et al. (2022) provides preliminary support for this link, showing that ESL learners with stronger academic self-concepts also report higher L2 self-confidence and lower anxiety.

Despite this, the literature also highlights variability based on individual and contextual factors. Students’ L2 self-confidence may not always rise in tandem with ASC, particularly if language-specific challenges, prior negative experiences, or social pressures undermine their communicative self-perceptions (Peng, 2016). This suggests that while ASC can facilitate the development of L2 self-confidence, the relationship is not automatic or unidirectional.

As such, further research is warranted, particularly in underexplored cultural contexts and among international learners, to elucidate how academic self-concept and L2 self-confidence interact over time, and to what extent interventions targeting ASC might enhance communicative confidence in second language settings.

### 2.4. Research Gap and Rationale

Despite growing recognition of the interplay among WTC, L2 self-confidence, and ASC, several important gaps remain in the current literature. First, much of the empirical research on these constructs has been conducted in classroom-based ESL contexts, often within learners’ home countries, and frequently with single-method, cross-sectional designs (e.g., Pattapong, 2015; Tam, 2022). As a result, within cross-sectional designs, there is limited understanding of how these variables interact in international academic environments, where learners must navigate unfamiliar linguistic, cultural, and social landscapes on a daily basis. It should be noted that the present study does not aim to make up for the lack of longitudinal scholarships.

For studies that do explore these constructs among international students, they often focus on broad measures of adaptation or proficiency, rather than specifically examining the dynamic relationships between ASC, self-confidence, and communicative behaviour (e.g., Lyons et al., 2014; Yue, 2016). Few investigations integrate both quantitative and qualitative approaches to provide nuanced insight into how learners perceive and respond to communication challenges in real-world academic settings.

Furthermore, there is a lack of research addressing how cultural factors, such as face-saving, collectivism, self-comparison, etc., moderate the effects of ASC and self-confidence on WTC, especially among learners from East and Southeast Asia studying Western English-first language countries. These factors are likely to influence not only self-perceptions but also learners’ willingness to engage in, or withdraw from, communicative opportunities. Finally, while theoretical models such as D. MacIntyre et al.’s (1998) heuristic model and Mercer’s (2011) work on academic self-concept offer valuable frameworks, there is a clear need for empirical studies that test and refine these models in multicultural, international higher education contexts.

Given these gaps, the present study adopts a mixed-methods approach to investigate the interrelationships among WTC, L2 self-confidence, and academic self-concept in Vietnamese university students studying in the United Kingdom. By integrating quantitative survey data and qualitative interviews, this research seeks to provide a richer, contextually grounded understanding of the psychological and behavioural factors shaping communicative perception in multicultural academic environments. The findings aim to extend existing theory and inform practical strategies for supporting international students’ academic and social success.

The present study, therefore, aims to answer the following research questions:

1. What are Vietnamese learners’ perceptions of WTC, academic self-concept, and L2 self-confidence?
2. What are the relationships among these three constructs?

## 3. Materials and Methods

### 3.1. Research Design

This study employed an explanatory sequential mixed-methods design (Creswell & Plano Clark, 2018), consisting of an initial quantitative phase followed by a qualitative phase. This approach was intentionally selected to address the research questions, i.e., *What are Vietnamese learners’ perceptions of willingness to communicate, academic self-concept, and L2 self-confidence? What are the relationships among these three constructs?*

The quantitative component provided a broad overview of participants’ self-reported levels of WTC, L2 self-confidence, and ASC, as well as the statistical relationships among these variables. This allowed for general patterns and associations to be identified across the cohort.

Subsequently, the qualitative component used in-depth interviews to further explore and contextualise these quantitative findings, offering nuanced insights into how learners experience, interpret, and navigate these constructs in real academic and social settings. In particular, the qualitative phase enabled the study to capture participants’ personal stories, perceived influences, and the lived complexity underlying their survey responses.

By integrating these two methodological strands, the study achieves both breadth and depth in examining the research questions. The sequential design ensured that qualitative inquiry was directly informed by and responsive to the quantitative results, providing a richer and more comprehensive understanding of the psychological and behavioural factors shaping Vietnamese students’ communication in an English-medium university context.

### 3.2. Ethical Consideration

The study was approved by the Human Research Ethics Committee of the University of Northampton. All participants provided informed consent and were assured of confidentiality, voluntary participation, and the right to withdraw at any time without consequence. Data were anonymized, securely stored, and managed in accordance with institutional and UK data protection regulations (BERA, 2018; GDPR, 2024; UON, 2024).

### 3.3. Participants and Sampling

Quantitative Phase: Participants were Vietnamese undergraduate and postgraduate students enrolled at a single public university in the United Kingdom. Eligibility criteria included (a) current full-time enrolment, (b) a Vietnamese cultural and linguistic background, and (c) at least one semester of study in an English-medium environment. A total of 32 students completed the online survey. Sampling combined purposive and convenience elements: recruitment was conducted through university mailing lists, student forums, and Vietnamese student association networks to maximise reach within this niche population. Although this approach limited the generalisability of findings, it was appropriate given the exploratory focus and the challenges of recruiting international student participants.

Qualitative Phase: From the survey respondents, five students were purposively and conveniently selected for follow-up semi-structured interviews, using maximum variation sampling to ensure diversity in gender, study program, and reported WTC levels. The students were recruited across five programs, both undergraduate and postgraduate: TESOL, Marketing, Accounting and Finance, and Fashion Design, which covered the larger previous quantitative cohort of participants. Albeit a small sample, this purposive subsample enabled exploration of both shared and divergent experiences. All interviewees provided additional written consent prior to participation. Participants’ demographic details for both phases are included in Table A1, Appendix B. 

### 3.4. Instrument

The quantitative survey included three standardized, validated scales adapted for the study context: WTC consists of 8 items from McCroskey & Baer (1985), targeting different social and academic interaction contexts; L2 Self-Confidence is comprised of 10 items, combining subscales for Self-Perceived Communicative Competence (SPCC; 4 items, P. D. MacIntyre & Charos, 1996) and Communication Anxiety (CA; 6 items, Lee & Lee, 2020), with composite scoring as per Clément (1980); and ASC consists of 5 items from the Academic Self-Concept Clarity Scale (Campbell et al., 1996), focused on English-medium academic tasks. All items used a five-point Likert scale (1 = strongly disagree, 5 = strongly agree). The survey was piloted with 10 Vietnamese students (not in the main sample) to ensure clarity and cultural appropriateness; minor language modifications were made following pilot feedback.

The qualitative interview schedule comprised 10 open-ended questions, adapted from Pattapong (2015), covering language background, perceptions of WTC, L2 self-confidence, ASC, and the contextual factors influencing these constructs. Interviews encouraged elaboration on both positive and challenging experiences, and on the perceived impact of social, cultural, and academic environments. Key mapping of survey/interview questions to the corresponding variables and research questions is included in Table A2, Appendix B. 

### 3.5. Data Collection

Survey data were collected online over four weeks, with reminders sent via university channels. Participation was voluntary, anonymous, and uncompensated.

Interviews were conducted individually via video call or in person, according to participant preference. The mixed model of interviews was optimized due to time constraints, i.e., the main interview phase happened over summer break, and most students were already leaving the country for home visits. No major differences in participants’ engagement were observed across models. The interviews were conducted in English, acknowledging that participants’ prior language exposure and living abroad experience should make them communicate fully and comfortably.

Each session lasted 40–60 min and was audio-recorded with consent. Interviews were transcribed verbatim by the researcher, and all participants had the opportunity to review and clarify their transcripts (member checking) to enhance trustworthiness. No major modification was witnessed after the member checking procedure.

To ensure participant anonymity, all interview transcripts were de-identified, and each participant was assigned a pseudonym (“fake” name, e.g., Julie, Jennie, etc.) for use in reporting qualitative results; no identifying information was linked to the data at any stage of analysis or dissemination.

### 3.6. Data Analysis

Quantitatively, descriptive statistics (means, standard deviations) were calculated for each scale. Internal consistency was assessed using Cronbach’s alpha (WTC: α = 0.72, SPCC: α = 0.70, CA: α = 0.88, ASC: α = 0.60 after item reduction and inter-correlation valid at 0.28). Given non-normality and the modest sample size, Kendall’s tau-b correlation coefficients were used to examine relationships among the three constructs and their subscales.

Qualitatively, transcripts were analysed thematically using the Braun et al. (2019) approach, facilitated by qualitative data analysis software (Delve via delvetool.com). Coding was deductive, guided by the study’s conceptual framework, but remained open to emergent themes reflecting participants’ unique experiences. Coding proceeded in three stages (Cohen et al., 2007): initial open coding, categorisation into higher-order themes (e.g., WTC context, confidence trajectory, and self-concept shifts), and synthesis with quantitative findings. To strengthen credibility, a secondary researcher reviewed a sample of coded transcripts, and coding decisions were discussed until consensus was reached.

Following independent analysis, quantitative and qualitative findings were integrated in the interpretation phase, using a side-by-side comparison to identify convergence, complementarity, and divergence. This allowed for a comprehensive, triangulated understanding of how WTC, L2 self-confidence, and ASC manifest in this population.

## 4. Results

This section presents the findings from both the quantitative and qualitative phases of the study. Results are organized by construct (WTC, L2 self-confidence, and ASC) and by phase (quantitative and qualitative), followed by a summary of the correlational analysis. Quantitative scores in this section should only be treated as participants’ perceptions rather than a direct measurement of participants’ proficiency or measurable behaviours.

### 4.1. Quantitative Results

#### 4.1.1. Descriptive Statistics

Descriptive analyses were conducted to characterise participants’ self-reported levels of willingness to communicate (WTC), L2 self-confidence (L2SC), and academic self-concept (ASC). Prior to analysis, internal consistency of the scales was assessed using Cronbach’s alpha and found to be satisfactory for all measures (WTC: α = 0.72; SPCC: α = 0.70; CA: α = 0.88; ASC: α = 0.60 after exclusion of two items with valid inter-correlation at 0.28; see Table A3, Table A4, Table A5, Table A6 and Table A7, Appendix B).

Overall, students reported moderate WTC (M = 3.50, SD = 0.59), slightly below-medium L2 self-confidence (M = 3.17, SD = 0.73), and moderate academic self-concept (M = 3.02, SD = 0.48) (Table A8, Appendix B). Examination of item-level data (Table A9, Appendix B) indicated that the majority of participants expressed a strong willingness to communicate in diverse groups (M = 4.00, SD = 0.88; 96%) and were comfortable discussing freely in English when acquainted with someone in the group (M = 3.81, SD = 0.85; 90%). Conversely, willingness to engage decreased in unfamiliar settings or with strangers (M = 2.94, SD = 1.01; M = 3.25, SD = 1.19, respectively), with less than one-third of students reporting active engagement under these conditions.

Aggregate L2SC, computed from SPCC and CA subscales, showed a medium mean score (M = 3.17, SD = 0.73), underpinned by a higher level of self-perceived communicative competence (M = 3.58, SD = 0.77) and a relatively lower level of communication anxiety (M = 2.76, SD = 0.94). Students reported feeling most confident when communicating in English with foreign friends (M = 3.78, SD = 1.09) or within small groups (M = 3.66, SD = 1.06). Despite this, a notable proportion of students reported experiencing anxiety in situations involving L2 task performance (M = 3.31, SD = 1.23) or expressing complex ideas (M = 3.34, SD = 1.18) (Table A10, Table A11 and Table A12, Appendix B).

For academic self-concept, mean scores reflected a moderate self-assessment (M = 3.02, SD = 0.48), with most participants indicating a clear and stable self-image (M = 2.67, SD = 1.09) and clarity in their academic goals (M = 2.97, SD = 1.16) (Table A13, Appendix B).

#### 4.1.2. Correlational Analysis

To examine the relationships among the three primary constructs, Kendall’s tau-b correlations were employed due to the modest sample size and non-normal distribution of data. The results in Table 1 below provide clear evidence of significant associations between the variables.

A significant, moderate positive correlation was observed between WTC and self-perceived communicative competence (SPCC) (τb = 0.46, *p* < 0.01). WTC was also negatively, though non-significantly, correlated with communication anxiety (CA) (τb = −0.24, *p* > 0.05). When considering the composite L2SC score, a significant, moderate positive association was found with WTC (τb = 0.39, *p* < 0.01), indicating that greater self-confidence is linked to increased willingness to communicate. The inverse relationship with anxiety suggests that lower anxiety may support higher WTC, but the evidence here was not statistically robust.

There was also a significant, moderate positive correlation between L2SC and ASC (τb = 0.26, *p* = 0.05). This relationship was mirrored in a significant, moderate negative correlation between CA and ASC (τb = −0.27, *p* < 0.05), while the association between SPCC and ASC was positive but did not reach significance (τb = 0.20, *p* > 0.05). Lastly, a weak, non-significant positive correlation was found between WTC and ASC (τb = 0.11, *p* > 0.05).

**Figure 1:**
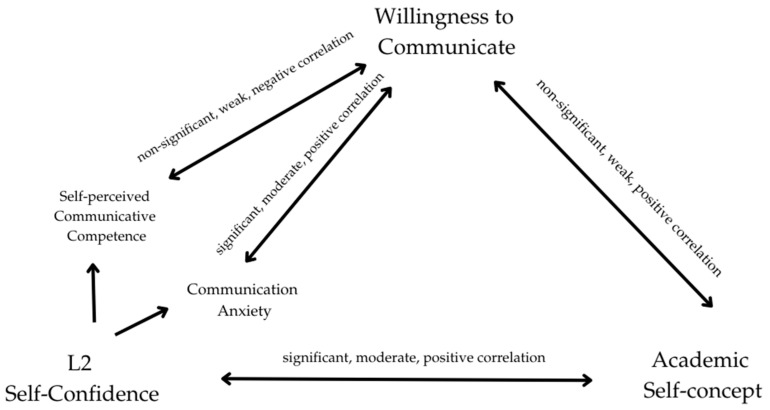
Visual display of correlational analysis.

Taken together, these findings suggest that L2 self-confidence serves as a key bridge between students’ academic self-concept and their communicative engagement, with anxiety and context exerting additional, albeit more variable, influence. The overall correlational analysis among the three factors therefore displayed in Figure 1 below:

### 4.2. Qualitative Findings

#### 4.2.1. Willingness to Communicate

Students being interviewed reported that WTC is their main reason for learning English, especially in cultural exchange and broadening their network. For example, Jennie emphasized being able to use English in real life excited her the most, while Julie highly rated learning cultural values by talking to people.

*“I’d say I enjoy speaking and communicating in English. Maybe my opinion is different but it’s not about theoretical things like grammar or vocabulary, it’s about practical aspects, it’s the fact that I can use English in real life”*

(Jennie)

*“I cannot tell why, but when I use English, whether it is at school or outside to talk to someone one I’ve ever known. I think it’s a great way to know more about people from other cultures, just by talking to them and learning something you’ve never experienced. I think I feel very special when I can speak English like that”*

(Julie)

Moreover, students preferred to communicate in small groups, especially with those familiar to them, like friends or acquaintances. It was the supportive environment and atmosphere of being with friends that encouraged the students to express themselves more freely.

*“I feel safe in small group. They’re my friends, so they don’t judge me”*

(Julie)

*“When we talk, we learn from each other. We feel supported in that group”*

(Ami)

Although the majority of students enjoy L2 communication, one student, Halie, admitted to only using English for academic contexts while avoiding communication outside the classroom. She explained her struggle as “not knowing how to connect and interact with people from diverse backgrounds” (Halie), emphasized that even if she initiated the conversation, it would shortly end.

*“There were times I tried to talk to someone, but the conversation ended soon … I just said hi and asked some personal information about them … After a while, I didn’t know what more to ask, and they didn’t ask me further …”*

(Halie)

#### 4.2.2. L2 Self-Confidence

Students’ confidence level ranged from medium to high, rooted either in their personality or positively affected by their surrounding environment, i.e., studying abroad. Students were confident handling daily conversation, expressing opinions, and reflecting a sense of “being” in conversation engagement (Ami, Jennie)

*“One thing I think about is how to get my perspectives and really ‘be” in the conversations. I would say when I want to speak, I would try with all I can to tell you whatever works for the topic I’m delivering”*

(Jennie)

Specifically, Julie and Ami confessed their confidence grew over time due to a supportive study environment in the UK.

*“I would say I was not very confident the first time I was here. Through time, I feel like I gain some respect communicating in class, thus I gain more confidence”*

(Julie)

*“I think it’s the environment the teacher created in the class. I feel like nobody will judge my English skills as everybody just encourages you to be yourself”*

(Ami)

All students viewed themselves as fluent and advanced speakers, aligning with the quantitative results. Competence was often described as the result of long-term learning, rather than something newly acquired.

*“It’s an ongoing process, I think I’m quite good at speaking now… I can make jokes too … now I can just speak naturally.”*

(Ami)

Despite high self-ratings, students preferred informal contexts. Halie, for example, felt more comfortable in small groups but reported difficulty with formal situations like presentations, which suggests the rise in communication anxiety leads to the decay of competence.

*“In a casual chat, I bring myself into the conversation… but in serious contexts, I get nervous and can’t think clearly.”*

(Halie)

Most students shared that they had previously struggled with a fear of making mistakes but had since developed a more accepting mindset. For example:

*“I used to fear others would make fun of me… but now I think it’s natural.”*

(Julie)

*“Before, I wasn’t confident when I made mistakes… now I just embrace it as something to work on.”*

(Mie)

However, anxiety still affected some students, especially in formal settings. Halie described intense nervousness during presentations, leading to self-consciousness and disrupted fluency.

*“When everyone looks at me, I feel like my brain stops… I’m scared they won’t understand me.”*

(Halie)

In such cases, anxiety appeared to interfere with performance, despite relatively strong underlying competence.

#### 4.2.3. Academic Self-Concept

In conversations, learners depicted themselves as having an active, flexible and open self-concept. For example:

*“I think mine should be friendly, curious and adaptable … I’ve just decided to be open and responsive to different styles of communication”*

(Julie)

*“.... Having quite an adaptable personality helped me a lot in communicating, building up dialogue and conversations with other people”*

(Jennie)

However, some students are affected by self-comparison, especially when they live and study in an English-speaking environment, which causes the instability of self-concept. However, students were well aware and embraced it as a process of self-improvement, as captured in this quote:

*“There will be times I feel like I am no good, it happens all the time, but I think it’s the way you face it”*

(Jennie)

What is more interesting is that in the case of Ami, the self-comparison came especially when she communicated with other Vietnamese colleagues, feeling they were secretly judging her speaking skills. However, she emphasized it was only her self-awareness; therefore, it would not prevent her from communicating.

*“… It’s just a kind of feeling that when I speak English, I feel like some Vietnamese classmates will judge my English. I feel they know my culture, where I come from, and I keep comparing myself… I mean, it still happens, but it does not mean I will stop talking”*

(Ami)

It should be noted that social comparisons may be something these students still face, though it is more likely to be an observation only and does not reduce the students’ confidence or destroy their self-concepts. Participants seemed well aware of the comparisons, yet they accepted and moved on from them, as in the case of Jennie, who asserted not paying much attention to them every now and then.

*“I know that they are better than me. It happens sometimes, but I just don’t pay much attention to it, rather on what I can do and can be now. I think in that way it’s more of a motivation for me to learn more and be a better me”*

(Jennie)

The data above proved that despite the comparisons, the students were still somewhat aware of their academic self-concept by having a healthy image of themselves and knowing what they want to become, which, again, is in line with the previous quantitative data recorded.

## 5. Discussion

### 5.1. Summary of Main Findings

This study investigated the interrelationship among WTC, L2 self-confidence, and academic self-concept in Vietnamese university students studying in the United Kingdom, employing an explanatory sequential mixed-methods design. The quantitative findings revealed that participants generally reported moderate levels of WTC and L2 self-confidence, and a fair but somewhat variable academic self-concept. Significant positive correlations were identified between WTC and L2 self-confidence, and between L2 self-confidence and academic self-concept, while the association between WTC and academic self-concept was positive but weak and not statistically significant.

Qualitative insights further contextualized these results. Students perceived an enhancement in their willingness to communicate in supportive and familiar environments, especially when engaging with friends or acquaintances, and were often motivated by desires for cultural exchange and information sharing. L2 self-confidence was found to be dynamic: shaped by cumulative learning experiences, supportive classroom climates, and evolving personal mindsets toward communication anxiety and mistake-making. While most participants viewed themselves as competent and resilient in daily interactions, many reported persistent anxiety in formal or high-stakes situations, particularly when required to use academic language or present in public. Academic self-concept, though generally positive, was susceptible to fluctuations due to self-comparison and cultural expectations, especially when interacting with peers from similar backgrounds.

Overall, the findings indicate that L2 self-confidence plays a pivotal mediating role, linking learners’ internal self-concept to their observable communication behaviours. The results also highlight the influence of social context, personal agency, and cultural factors on the communication patterns of international students in English-medium academic environments.

### 5.2. Contextualising the Results Within Current Scholarship

The findings of this study both reinforce and extend existing understandings of WTC, L2 self-confidence, and academic self-concept within the field of second language acquisition and behavioural science. Taken together, the results offer several important insights into how psychological and contextual variables interact to shape the communicative behaviour of international students in multicultural academic settings. However, consideration should be made as there is a lack of direct factor measurement and observation in the current study, which should be treated perception-wise only.

The moderate levels of WTC reported align with previous research highlighting the importance of supportive and immersive environments in fostering communication (Ghonsooly et al., 2014; Katiandagho & Sengkey, 2022). While earlier studies in Asian EFL contexts have sometimes emphasized passivity, reticence, or “face-protective” behaviour among Asian learners (Tam, 2022), the current study demonstrates that immersion in an English-speaking environment and the necessity of using English for academic and social survival appear to outweigh many traditional cultural inhibitions. Students reported a willingness to communicate, particularly in small-group or familiar contexts, which echoes the findings of Ducker (2021) regarding the influence of group dynamics and social safety on WTC. Notably, even where apprehensions about negative evaluation or “being judged” persisted, especially in interactions with other Vietnamese students, these factors rarely resulted in avoidance or communicative withdrawal. This suggests an emerging resilience and adaptability among international learners, potentially fostered by exposure to diverse intercultural interactions and a shifting mindset towards language learning (Lou & Noels, 2020).

The dynamic nature of L2 self-confidence observed in this study resonates with the heuristic model proposed by D. MacIntyre et al. (1998), where self-confidence is conceptualized as a composite of self-perceived communicative competence and communication anxiety. Consistent with Ghonsooly et al. (2014), Fernando (2022), and Yashima (2002), participants in this study identified supportive classroom climates and positive interpersonal experiences as critical contributors to their self-confidence, particularly in informal contexts. The persistence of anxiety in formal or evaluative settings, such as presentations, underscores the enduring impact of situational variables on communicative behaviour. However, the willingness of students to reinterpret anxiety and mistake-making as natural and constructive elements of the learning process reflects a positive orientation toward language development: an orientation that may be linked to academic mindsets emphasising growth, resilience, and self-efficacy (Mercer, 2011). Notably, participants in this study did not report the debilitating self-doubt sometimes found in other samples (e.g., Zhao & Chang, 2022), suggesting that contextual and institutional factors, such as inclusive teaching practices and peer support, may mitigate the negative effects of communication anxiety.

Academic self-concept emerged in this study as a nuanced, contextually responsive construct. While self-comparison and cultural expectations introduced elements of instability and vulnerability, most participants demonstrated awareness of these influences and reframed them as sources of motivation rather than impediments. This aligns with previous work by Pattapong (2015), who emphasized the role of self-comparison in shaping learners’ academic identities and behavioural choices. The practice of “saving face” and striving for humility, often associated with Confucian-influenced educational cultures (Leung, 1998; Phuong-Mai, 2008), was present, but was typically balanced by a proactive and agentic stance toward language use and self-improvement. Interestingly, the findings indicate that academic self-concept, while influential, does not operate as a fixed personality trait but as a flexible and dynamic self-presentation shaped by the desire to communicate and succeed in new cultural contexts.

The interrelationships among WTC, L2 self-confidence, and academic self-concept reported in this study lend empirical support to the theoretical proposition that self-confidence mediates the influence of academic self-concept on communicative behaviour (D. MacIntyre et al., 1998; Mercer, 2011; Lubis et al., 2022). Quantitative analysis demonstrated that L2 self-confidence is a significant predictor of WTC, corroborating the work of Fatima et al. (2020) and Peng and Woodrow (2010). The moderate association between academic self-concept and L2 self-confidence further reinforces the idea that learners’ beliefs about their academic abilities underpin their communicative self-assurance, particularly in challenging or high-stakes environments. The positive, albeit non-significant, relationship between academic self-concept and WTC may reflect the complex and sometimes competing pressures faced by international students: while positive self-concept encourages engagement, ongoing self-comparison and cultural adaptation processes may introduce hesitation or fluctuation in communicative behaviours.

From a broader behavioural science perspective, the findings illustrate how psychosocial variables, such as perceived competence, anxiety, and self-concept, are not merely internal states but are dynamically co-constructed in relation to social context, cultural identity, and learning environment (Basarkod et al., 2024; Zhou et al., 2025). The nuanced interplay among these constructs in this cohort of Vietnamese students underscores the need for a holistic, context-sensitive approach to understanding language learning behaviour in multicultural higher education. These results not only affirm key tenets of the WTC and self-concept models but also highlight the importance of resilience, agency, and adaptability in the academic and social success of international learners.

### 5.3. Implications for International Language Education

The findings of this study offer several important implications for both theory and practice in the fields of second language acquisition, behavioural science, and international education.

This research advances the theoretical understanding of L2 communication by confirming and extending the heuristic model of WTC (D. MacIntyre et al., 1998) and related frameworks (Mercer, 2011; Bandura, 1986). It substantiates the role of L2 self-confidence as a central mediator between academic self-concept and communication behaviour and demonstrates that self-perceptions are not static personality traits, but dynamic constructs shaped by context, social interaction, and cultural adaptation. The finding that academic self-concept does not directly predict WTC, but operates in conjunction with self-confidence, refines our understanding of motivational and affective variables in L2 engagement. Moreover, the observed flexibility and agentic self-positioning among Vietnamese students in the UK points to the importance of viewing communicative behaviour as both context-sensitive and influenced by evolving self-beliefs. This holistic perspective enriches existing models by foregrounding the interplay of psychological, cultural, and situational variables in multilingual academic environments.

The study also yields actionable recommendations for educators, university administrators, and support professionals working with international and culturally and linguistically diverse (CALD) students. For educators, the results underscore the value of creating supportive, inclusive classroom environments that encourage risk-taking and foster communicative confidence. Strategies such as collaborative group work, peer mentoring, and positive feedback can help reduce communication anxiety and promote a growth mindset (Springer et al., 2025). Recognising that self-confidence and self-concept are malleable (Van der Aar et al., 2022), instructors should explicitly address these constructs through reflective activities, opportunities for safe practice, and discussions that normalise anxiety and mistake-making as part of the learning process.

For institutional policy and student support services, the findings highlight the need for targeted interventions that address the psychosocial as well as linguistic needs of international students. Orientation programs, counselling, and language support services should be designed to help students navigate cultural adjustment, manage self-comparison, and build resilience (Sakız & Jencius, 2024). Providing opportunities for meaningful intercultural interaction, both within and beyond the classroom, may further enhance willingness to communicate and academic self-concept (D’Orazzi & Marangell, 2025; Vromans et al., 2023).

From a clinical perspective, the study’s behavioural framing of communication, self-confidence, and self-concept may inform the design of support programs for students experiencing language-related anxiety or social withdrawal (Beesdo et al., 2009; Wilmot et al., 2024; Zhang & Zhang, 2022). Clinicians working with CALD or international students should assess not only linguistic proficiency but also underlying psychosocial variables (Khawaja & Wotherspoon, 2022), therefore, offering interventions that build communicative self-assurance and address anxiety in high-stakes or unfamiliar settings. In this sense, these findings also have broader implications for students from CALD backgrounds, particularly in relation to the acquisition and use of second language forms (as opposed to content or use; see Bloom & Lahey, 1978), e.g., morpho-syntax between typologically different L1s and L2s (e.g., Furukawa & Nakamura, 2024; Han, 2013; Han & Shi, 2016; Hwang & Steinhauer, 2011), where self-confidence and academic self-concept can significantly influence learners’ willingness to take communicative risks and experiment with more complex grammatical forms in authentic settings.

In summary, this research highlights the interdependence of psychological, behavioural, and contextual factors in shaping L2 communication. By adopting a holistic and context-sensitive approach, educators and support professionals can more effectively foster communicative engagement, confidence, and well-being among international students in higher education.

### 5.4. Limitations and Directions for Future Research

While this study provides valuable insights into the interplay between willingness to communicate, L2 self-confidence, and academic self-concept among Vietnamese university students in the United Kingdom, several limitations should be acknowledged. These constraints offer important context for interpreting the findings and highlight avenues for future research.

The relatively small sample size and the focus on students from a single university limit the generalisability of the results. The small scale of the qualitative sample, for example, can narrow down the study’s applicability. Also, the single-university setting created a unique profile that yielded desirable bias for replication. Although the mixed-methods approach allowed for in-depth exploration and triangulation of constructs, larger and more diverse samples, including students from multiple institutions, degree programs, and countries, would provide a broader basis for understanding these phenomena across different cultural and academic contexts.

Consequently, the study’s findings are embedded in the specific cultural, linguistic, and institutional context of Vietnamese students studying in the UK. Communication behaviours, self-confidence, and academic self-concept are influenced by a range of cultural and contextual variables, including prior language experiences, exposure to English, and institutional support structures. Replicating and extending this research among other international student groups, as well as in different host countries or educational settings, would allow for meaningful cross-cultural comparison and deepen our understanding of context-sensitive behavioural dynamics.

Further, the reliance on self-report questionnaires and semi-structured interviews may introduce bias, such as social desirability effects or recall bias. While the combination of quantitative and qualitative data strengthens the study, future research could benefit from incorporating additional methods such as classroom observation, behavioural tracking, or longitudinal designs. For example, following students across time points would shed light on how willingness to communicate, self-confidence, and academic self-concept evolve in response to new challenges, achievements, and social interactions.

While this study focused on the interrelationships among three core constructs, additional variables such as motivation, language proficiency, social network size, or perceived support were not systematically examined. Future research could explore how these and other factors interact with WTC, self-confidence, and academic self-concept, potentially revealing more complex or indirect pathways influencing language behaviour. Additionally, future work should seek to design, implement, and assess targeted interventions, such as workshops on communication confidence, peer mentoring schemes, or culturally responsive counselling, to determine their impact on students’ behavioural engagement and well-being.

While acknowledging its limitations, this study contributes to a growing evidence base on the behavioural and psychological determinants of communicative success among international learners. Addressing the above constraints in future research will help to refine theoretical models, inform context-sensitive practice, and support the academic and social integration of culturally and linguistically diverse students in higher education.

## 6. Conclusions

This study provides new insights into the complex interplay between willingness to communicate, L2 self-confidence, and academic self-concept among Vietnamese university students navigating English-medium education in the United Kingdom. By integrating quantitative and qualitative data, the research demonstrates that L2 self-confidence is a crucial mediator connecting students’ internal self-perceptions to their observable communication behaviours, and that supportive social and academic environments can empower learners to move beyond traditional cultural inhibitions.

The findings advance theoretical understanding of language learning by refining the heuristic model of WTC and emphasising the dynamic, context-dependent nature of communicative behaviour in multicultural settings. Practically, the study points to the importance of fostering environments that build confidence, embrace anxiety as part of learning, and support positive self-concept, key strategies for educators, support staff, and clinicians working with international and CALD students.

As international student mobility and linguistic diversity continue to shape global higher education, this research underscores the need for holistic, context-sensitive approaches that support the psychological and behavioural well-being of learners. By shedding light on the factors that facilitate communicative engagement and resilience, this study offers both a foundation for future research and practical guidance for enhancing academic and social success in multicultural classrooms.

## Figures and Tables

**Table 1:** Correlation among Willingness to Communicate, L2 Self-Confidence and Academic Self-Concept.

		WTC	SPCC	CA		SC	ASC
Kendall’s tau_b	Willingness to Communicate (WTC)	Correlation Coefficient	1.000	0.469	−0.242	0.389	0.112
		Sig. (2-tailed)	.	0.000	0.069	0.003	0.407
	Self-perceived Communicative competence (SPCC)	Correlation Coefficient	0.469	1.000	−0.165	0.644	0.199
		Sig. (2-tailed)	0.000	.	0.216	0.000	0.140
	Communication Anxiety (CA)	Correlation Coefficient	−0.242	−0.165	1.000	−0.565	−0.266
		Sig. (2-tailed)	0.069	0.216	.	0.000	0.048
	L2 Self-Confidence (SC)	Correlation Coefficient	0.389	0.644	−0.565	1.000	0.257
		Sig. (2-tailed)	0.003	0.000	0.000	.	0.050
	Academic Self-Concept (ASC)	Correlation Coefficient	0.112	0.199	−0.266	0.257	1.000
		Sig. (2-tailed)	0.407	0.140	0.048	0.050	.
c. Listwise N = 32							

## Data Availability

The raw data supporting the conclusions of this article will be made available by the authors on request.

## Abbreviations

The following abbreviations are used in this manuscript:

ASC	Academic Self-Concept
CA	Communication Anxiety
L2SC	L2 Self-Confidence
SPCC	Self-perceived Communicative Competence
WTC	Willingness to Communicate

## Appendix B

### Appendix B.1. Demographic Details of Participants and Key Variables of Qualitative and Quantitative Phases

**Table A1:** Demographic details of participants.

Factor	Quantitative Phase (Survey)	Qualitative Phase (Interview)
Age	18–30	18–30
Gender	15 males, 17 females	5 females
Degree	Undergraduate and Postgraduate (Master’s)	Undergraduate and Postgraduate (Master’s)
Program	Fashion Design BSc, Global Business Management Top-up BA, TESOL MA, MSc Marketing, MSc Accounting and Finance	Fashion Design BSc, Global Business Management Top-up BA, TESOL MA, MSc Marketing, MSc Accounting and Finance (1 student/major)
Years in the UK	6 months–5 years	6 months–5 years

**Table A2:** Key variables across survey, interview, and research questions.

Variables	Sub-Variables	Survey Question Number	Interview Question Number	Research Question 1	Research Question 2
Willingness to Communicate		1–8	10	Survey + Interview questions	Survey question
L2 Self-Confidence	Self-perceived Communicative Competence	9–12	4–6		
	Communication Anxiety	13–18			
Academic Self-Concept		19–23	7–9		

### Appendix B.2. Cronbach’s Alpha Level of Variables and/or Inter-Correlation Between Items

**Table A3:** Cronbach’s alpha level of Willingness to Communicate.

Cronbach’s Alpha	Cronbach’s Alpha Based on Standardized Items	N of Items
0.729	0.737	8

**Table A4:** Cronbach’s alpha level of Self-perceived Communicative Competence.

Cronbach’s Alpha	Cronbach’s Alpha Based on Standardized Items	N of Items
0.694	0.695	4

**Table A5:** Cronbach’s alpha level of Communication Anxiety.

Cronbach’s Alpha	Cronbach’s Alpha Based on Standardized Items	N of Items
0.880	0.881	6

**Table A6:** Cronbach’s alpha level of Academic Self-Concept.

Cronbach’s Alpha	Cronbach’s Alpha Based on Standardized Items	N of Items
0.592	0.547	3

**Table A7:** Cronbach’s alpha level of intercorrelation between items.

	Mean	Minimum	Maximum	Range	Maximum/Minimum	Variance	N of Items
Item Means	2.908	2.621	3.172	0.552	1.211	0.076	3
Inter-Item Correlations	0.287	0.120	0.457	0.337	3.807	0.023	3

### Appendix B.3. Descriptive Statistics of Variables

**Table A8:** Descriptive statistics of Willingness to Communicate, L2 Self-Confidence, and Academic Self-Concept.

	N	Mean	Std. Deviation
Willingness to Communicate	32	3.5000	0.59906
L2 Self-Confidence	32	3.1758	0.73687
Academic Self-Concept	32	3.0219	0.48824
Valid N (listwise)	32		

**Table A9:** Descriptive statistics of Willingness to Communicate.

	N	Mean	Std. Deviation
Initiate a conversation with a classmate	32	3.34	1.125
Talk freely in English in a group/in-class discussion with people (you know well some of them)	32	3.81	0.859
Talk freely in English in a group/in-class discussion with people (you do not know them well)	32	3.53	1.016
Present your idea publicly in front of others	32	3.56	1.076
Talk to a stranger (e.g., tourist) in English in a public place (bus stop, train station, etc.)	32	3.25	1.191
Join in a conversation in English with a group of people from other national backgrounds (you know some of them)	32	4.00	0.880
Join in a conversation in English with a group of friends from other national backgrounds (it is your first time with them)	32	3.56	0.948
Talk freely in English in a large meeting of strangers	32	2.94	1.014
Valid N (listwise)	32		

**Table A10:** Descriptive statistics of L2 Self-Confidence.

	N	Minimum	Maximum	Mean	Std. Deviation
Communication Anxiety (reverse code)	32	1.67	5.00	2.7656	0.94552
L2 Self-Confidence	32	1.63	4.88	3.1758	0.73687
Self-perceived communicative competence	32	1.25	5.00	3.5859	0.77929
Valid N (listwise)	32				

**Table A11:** Descriptive statistics of Self-Perceived Communicative Competence.

	N	Mean	Std. Deviation
Talk in English to a foreign friend	32	3.78	1.099
Talk in English to a foreign stranger	32	3.50	1.047
Talk in English to a small group of people (you know some of them)	32	3.66	1.066
Talk in English to a small group of strangers	32	3.41	1.103
Valid N (listwise)	32		

**Table A12:** Descriptive statistics of Communication Anxiety.

	N	Mean	Std. Deviation
I feel nervous when I speak in English in front of other students	32	3.25	1.107
I feel anxious if I am asked a question in English by my teacher	32	3.25	1.295
When speaking in English, I can get so nervous that I forget things that I know	32	3.34	1.181
I feel nervous when I am called upon to perform a task in English	32	3.31	1.230
Even if I am well prepared for English class, I feel anxious about it	32	3.25	1.136
I am afraid that the other students will laugh at me when I speak English	32	3.00	1.218
Valid N (listwise)	32		

**Table A13:** Descriptive Statistics of Academic Self-Concept.

	N	Mean	Std. Deviation
My belief about myself often conflicts with that of others	30	2.67	1.093
In general, I do not have a clear sense of who I am and what I am	32	3.19	1.061
It is often hard for me to make up my mind about things because I do not really know what I want	31	2.97	1.169
Valid N (listwise)	29		
